# In-Human Multiyear Evolution of Carbapenem-Resistant Klebsiella pneumoniae Causing Chronic Colonization and Intermittent Urinary Tract Infections: A Case Study

**DOI:** 10.1128/msphere.00190-22

**Published:** 2022-05-09

**Authors:** Michelle Kalu, Karen Tan, Marquerita Algorri, Peter Jorth, Annie Wong-Beringer

**Affiliations:** a University of Southern California, School of Pharmacy, Los Angeles, California, USA; b Cedars-Sinai Medical Center, Department of Pathology and Laboratory Medicine, Los Angeles, California, USA; c Cedars-Sinai Medical Center, Department of Medicine, Los Angeles, California, USA; d Cedars-Sinai Medical Center, Department of Biomedical Sciences, Los Angeles, California, USA; e Huntington Memorial Hospital, Department of Pharmacy Services, Pasadena, California, USA; CDC

**Keywords:** *Klebsiella pneumoniae*, biofilms, carbapenems, evolution, urinary tract infection

## Abstract

Carbapenem-resistant Klebsiella pneumoniae (CRKP) is a frequent pathogen of the urinary tract, but how CRKP adapts *in vivo* over time is unclear. We examined 10 CRKP strains from a patient who experienced chronic colonization and recurrent urinary tract infections over a period of 4.5 years. We performed whole-genome sequencing and phenotypic assays to compare isolates that had evolved relative to the first isolate collected and to correlate genetic and phenotypic changes over time with the meropenem-containing regimen received. Phylogenetic analysis indicated that all 10 strains originated from the same sequence type 258 (ST258) clone and that three sublineages (SL) evolved over time; strains from two dominant sublineages were selected for detailed analysis. Up to 60 new mutations were acquired progressively in genes related to antibiotic resistance, cell metabolism, and biofilm production over time. Doubling of meropenem MICs, increases in biofilm production and *bla*_KPC_ expression, and altered carbon metabolism occurred in the latter strains from the last sublineage compared to the initial strain. Subinhibitory meropenem exposure *in vitro* significantly induced or maintained high levels of biofilm production in colonizing isolates, but isolates causing infection were unaffected. Despite acquiring different mutations that affect carbon metabolism, overall carbon utilization was maintained across different strains. Together, these data showed that isolated urinary CRKP evolved through multiple adaptations affecting carbon metabolism, carbapenem resistance, and biofilm production to support chronic colonization and intermittent urinary tract infections. Our findings highlight the pliability of CRKP in adapting to repeated antibiotic exposure and should be considered when developing novel therapeutic and stewardship strategies.

**IMPORTANCE** Carbapenem-resistant Klebsiella pneumoniae (CRKP) can cause a variety of infections such as recurrent urinary tract infections (rUTI) with the ability to change with the host environment over time. However, it is unclear how CRKP adapts to the urinary tract during chronic infections and colonization. Here, we studied the evolution of CRKP strains from a patient who experienced chronic colonization and recurrent UTIs over a period of 4.5 years despite multiple treatment courses with meropenem-containing regimens. Our findings show the flexibility of CRKP strains in developing changes in carbapenem resistance, biofilm production, and carbon metabolism over time, which could facilitate their persistence in the human body for long periods of time in spite of repeated antibiotic therapy.

## INTRODUCTION

Carbapenem-resistant Klebsiella pneumoniae (CRKP) strains have emerged since the early 2000s and have spread globally (1). CRKP can cause a variety of infections, such as recurrent urinary tract infections (UTI), pneumonia, and bloodstream infections, with mortality rates as high as 70% for the latter (2, 3). A recent epidemiologic study from a consortium of 18 health care systems in the Great Lakes region of the United States indicated that urine is the predominant source of CRKP (63%) (4). Among patients colonized with CRKP in the urinary tract, half presented with infection. Published literature on the genetic background of the circulating strains that cause UTIs indicates that majority of the isolates (75%) in the United States and globally belong to sequence type 258 (ST258) (5–7).

Colonization of CRKP at the anatomic site can lead to persistence of CRKP strains in the human body through attachment to epithelial cells, and persistence of CRKP in the gastrointestinal tracts of patients for as long as 5 years has been reported (8–11). These studies document mutations acquired over time in genes impacting metabolism, antibiotic resistance, and biofilm production on both plasmids and chromosomes of CRKP isolates. More specifically, CRKP strains can form biofilms which can protect against antibiotics and host immune responses, enhancing their ability to persist on epithelial cells (12–15). In addition, patients infected with biofilm-producing K. pneumoniae strains isolated from the blood and lungs can experience a shorter time to death compared to those infected with non-biofilm-producing K. pneumoniae strains (3 days versus 11 days), suggesting that infections with biofilm-producing strains can be more lethal (16). Biofilm-producing strains can infect the urethra and colonize the bladder, where bacterial structures such as fimbriae, capsule, and porins facilitate bacterial adherence to the bladder epithelium. Persistence of bacteria in the bladder can lead to recurrent urinary tract infections (1). These CRKP strains can also express Klebsiella pneumoniae carbapenemase (KPC) encoded by the *bla*_KPC_ gene to survive in the presence of carbapenem and cephalosporin therapy (13, 17). It is currently unknown how CRKP strains adapt over time in the urinary tract following repeated courses of antibiotic therapy. Here, we detail the in-human evolution of CRKP by characterizing the genetic mutations and phenotypic changes in multiple strains of CRKP ST258 obtained sequentially from a single patient who was chronically colonized with intermittent infections over 4.5 years and correlate these changes with the patient’s clinical course and antibiotic therapy.

## RESULTS

### Clinical course of patient case.

Patient X was a 78-year-old male with multiple comorbid conditions, including benign prostatic hyperplasia, who became chronically colonized with CRKP in the urine with intermittent infections over the course of 4.5 years. The patient was hospitalized 25 times during the 4.5-year interval and eventually expired in 2015 from bacteremia due to CRKP (see Table S1 in the supplemental material). Recurrent UTIs or colonization due to CRKP accounted for 84% (21/25) of hospital admissions. Overall, meropenem was most frequently prescribed during 20 (80%), followed by tigecycline during 9 (36%), and colistin during 6 (24%) of the 25 admissions. The patient had an indwelling urinary catheter throughout the 4.5 years which was changed during each hospital admission as part of standard care. The median length of hospital stay was 6 days (interquartile range [IQR], 3 to 7) with a median of 32 days (IQR, 13.75 to 66.25) between hospital admissions. The patient was discharged home from a majority of hospital admissions (88%, 22/25), except on 3 occasions in which the patient was transferred to another hospital, to an acute rehabilitation center for 1 month, and to a skilled nursing facility for 5 days, respectively.

10.1128/msphere.00190-22.2 TABLE S1 Complete description of all hospital stays and antibiotic therapy received by patient X. Download Table S1, DOCX file, 0.02 MB. Copyright © 2022 Kalu et al. 2022 Kalu et al. https://creativecommons.org/licenses/by/4.0/ This content is distributed under the terms of the Creative Commons Attribution 4.0 International license.

### WGS reveals coevolution of three sublineages from the same clonal origin over time.

**FIG 1:**
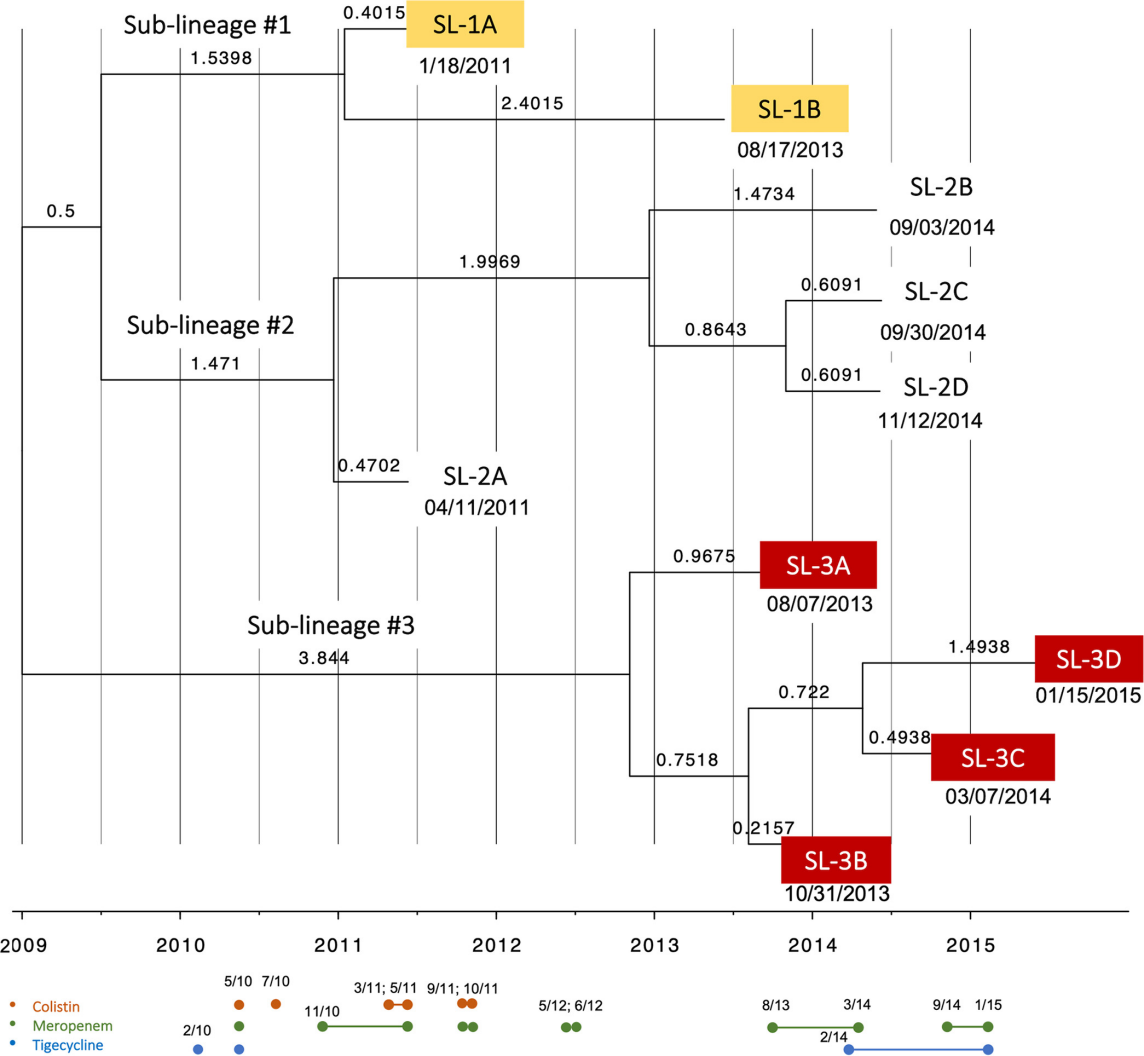
Three primary sublineages developed over time from the ancestral strain that infected patient X. Tree was constructed using single-nucleotide polymorphism (SNP) alignment and a time-scaled phylogeny approach (BEAST v1.10.4). Branch lengths indicate number of substitutions per site per year. Dates of isolation are written beneath isolate names. Colored circles below the tree represent the start and end of antibiotic treatment; colistin in orange, meropenem in green, tigecycline in blue; month/year of exposure is indicated. Sublineage 1 is highlighted in yellow; sublineage 3 highlighted in red.

Ten CRKP urinary strains that were obtained from patient X were confirmed to be clonally related by whole-genome sequencing (WGS). Multilocus sequence typing (MLST) analysis showed that the isolates belonged to the ST258 clonal group clade 2. A phylogenetic tree confirmed clonality of the strains, as there was a low number of substitutions per site close to the ancestral node relative to the reference isolate used to root the tree. The tree revealed the formation of two major clades among the 10 strains, one clade with sublineages 1 and 2 and one clade with sublineage 3 (Fig. 1). The first collected isolate, SL-1A (from 2011) and SL-1B (2013), formed sublineage 1. Sublineage 3 consisted of four isolates that persisted the longest in the patient (spanning 2013 to 2015) compared to sublineage 2, which included another four isolates from a single year, 2014. Of the 10 CRKP urinary isolates, those belonging to sublineage 1 were isolated when the patient was asymptomatic, while most strains from sublineages 2 and 3 (except for SL-2D and SL-3C) were associated with symptoms of infection. We selected strains from sublineages 1 and 3 to further explore the changes in carbapenem resistance, metabolism, and biofilm formation that occurred over time, since sublineage 1 included the earliest isolated strain and the terminal strains of sublineage 3 (SL-3C and SL-3D) persisted the longest in the patient from time of initial isolation.

Mutations occurring in the core genome were further analyzed, with a focus on genes related to metabolism, antibiotic resistance, and biofilm production (Table 1; see also Table S2). Compared to the reference strain AR_0113, all isolates shared approximately 69 mutations, which were present in the least-common ancestor that initiated the infection. The terminal strain SL-1B, isolated in 2013, acquired 47 new mutations over a 2.5-year period. In comparison, the terminal strains in sublineage 3 (SL-3C and SL-3D) acquired the largest numbers of mutations (*n* = 52 and 60, respectively), representing an accumulation of over 2.5-fold more mutations since the isolation of SL-1A in 2011.

**TABLE 1:** Summary of mutations in the 10 CRKP strains isolated from patient X^*a*^

Isolate	Date of Isolation	Infection/colonization	Total no. of mutations	No. of SNPs	No. of small indels	No. of large deletions	No. of nonsynonymous SNPs
SL-1A	18 January 2011	Colonization	93	78	12	3	22
SL-2A	11 April 2011	Infection	92	79	11	2	23
SL-3A	7 August 2013	Infection	109	92	15	2	26
SL-1B	17 August 2013	Colonization	116	101	12	3	24
SL-3B	31 October 2013	Infection	118	100	13	5	28
SL-3C	7 March 2014	Colonization	121	103	14	4	28
SL-2B	3 September 2014	Infection	101	87	12	2	23
SL-2C	30 September 2014	Infection	114	99	12	3	24
SL-2D	12 November 2014	Colonization	119	102	14	3	27
SL-3D	15 January 2015	Infection	129	107	16	6	30

a Genomic reads were mapped to reference strain AR_0113 using the *breseq* command line tool.

10.1128/msphere.00190-22.3 TABLE S2 Single-nucleotide polymorphism (SNP) distance matrix among the 10 carbapenem-resistant Klebsiella pneumoniae (CRKP) isolates isolated from patient X, calculated using the CSI Phylogeny 1.1a web server. Download Table S2, DOCX file, 0.01 MB. Copyright © 2022 Kalu et al. 2022 Kalu et al. https://creativecommons.org/licenses/by/4.0/ This content is distributed under the terms of the Creative Commons Attribution 4.0 International license.

### The evolution of antibiotic resistance in sublineages 1 and 3.

Differences in antibiotic resistance genes were observed among the strains as time progressed. All six strains carried *bla*_KPC-3_, *bla*_SHV-11_, and *bla*_TEM-1_ (Table 2). All strains except for SL-1A carried the *bla*_OXA-9_ gene. In addition, all six strains in sublineages 1 and 3 had a truncated *ompK35* gene. Only SL-1B had a partial deletion of the *ompK36* gene, which led to complete loss of the OmpK36 porin as confirmed by SDS-PAGE (Fig. S1). Loss of the OmpK35 and OmpK36 porins has been shown to contribute to significant increases in carbapenem MICs (18).

**TABLE 2:** Meropenem susceptibilities, beta-lactamase genes, and porin mutations present in sublineage 1 and 3 strains

Control or sublineage	Isolate	Infection/colonization	Meropenem MIC (ug/mL)	Beta-lactamase genes^*a*^	Porin mutation in:	
					*ompK35*	*ompK36*
Control	ATCC 25922		<4			
	ATCC 13883		<4			
	AR_0113		512	*bla*_KPC-3_, *bla*_SHV-11_	89* (frameshift mutation)	165* (frameshift mutation)
Sublineage 1	SL-1A	Colonization	32	*bla*_KPC-3_, *bla*_SHV-11_, *bla*_TEM-1_	89* (frameshift mutation)	
	SL-1B	Colonization	>1,024	*bla*_KPC-3_, *bla*_SHV-11_, *bla*_TEM-1_, ***bla*_OXA-9_**	89* (frameshift mutation)	Δ521 bp
Sublineage 3	SL-3A	Infection	64	*bla*_KPC-3_, *bla*_SHV-11_, *bla*_TEM-1_, ***bla*_OXA-9_**	89* (frameshift mutation)	
	SL-3B	Infection	48	*bla*_KPC-3_, *bla*_SHV-11_, *bla*_TEM-1_, ***bla*_OXA-9_**	89* (frameshift mutation)	
	SL-3C	Colonization	96	*bla*_KPC-3_, *bla*_SHV-11_, *bla*_TEM-1_, ***bla*_OXA-9_**	89* (frameshift mutation)	
	SL-3D	Infection	64	*bla*_KPC-3_, *bla*_SHV-11_, *bla*_TEM-1_, ***bla*_OXA-9_**	89* (frameshift mutation)	

a Bolded genes were acquired after the isolation of SL-1A in 2011.

10.1128/msphere.00190-22.4 FIG S1 Outer membrane porin SDS-PAGE for control strains and strains from sublineage 1 and 3 grown in high-osmolarity broth. Control strain (AR_0113) with confirmed porin truncations in OmpK35 and OmpK36; SL-1B highlighted in yellow. Download FIG S1, TIF file, 0.4 MB. Copyright © 2022 Kalu et al. 2022 Kalu et al. https://creativecommons.org/licenses/by/4.0/ This content is distributed under the terms of the Creative Commons Attribution 4.0 International license.

**FIG 2:**
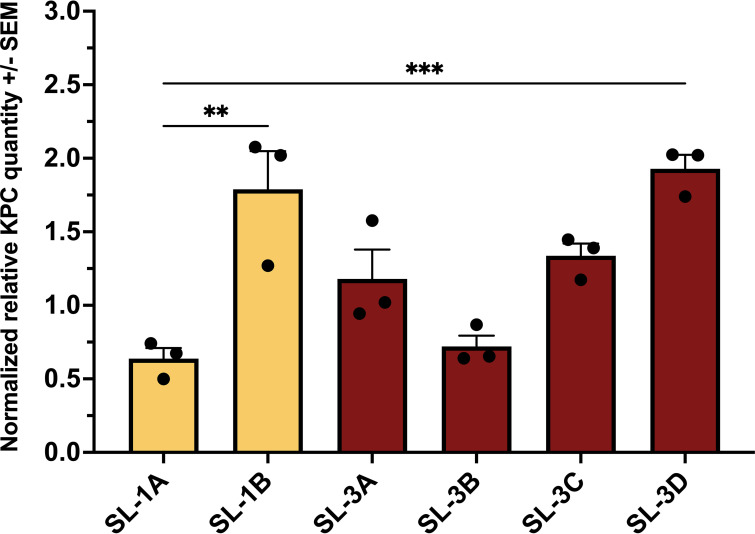
Baseline KPC expression in sublineages 1 and 3 over time. (A) KPC expression normalized to *repA* and *rpoD* reference genes. (B) Fold change relative to SL-1A. Sublineage 1 is highlighted in yellow; sublineage 3 is highlighted in red. Significance by one-way analysis of variance (ANOVA) (*, *P* < 0.05).

We examined if these genetic changes corresponded to phenotypic changes in antibiotic resistance, particularly meropenem resistance, since the patient received meropenem-containing regimens throughout the 4.5 years of infection. Broth microdilution MIC and quantitative reverse transcription-PCR (qRT-PCR) assays were performed to determine meropenem MICs and *bla*_KPC_ expression. Relative to the initial strain SL-1A, a trend toward a gradual increase in *bla*_KPC_ expression for strains isolated over time in sublineages 1 and 3 was observed, with the highest expression (a 2-fold increase relative to SL-1A) seen in the terminal strain, SL-3D, in sublineage 3 (Fig. 2). Interestingly, both SL-1A and SL-3B had the lowest *bla*_KPC_ expression. Following a similar pattern observed with *bla*_KPC_ expression, meropenem MICs for SL-1A and SL-3B were 32 μg/mL and 48 μg/mL compared to the elevated MICs at 96 μg/mL and 64 μg/mL for SL-3C and SL-3D (Table 2). Notably, the terminal strain of sublineage 1, SL-1B, had the highest meropenem resistance at an MIC of >1,024 μg/mL, most likely due to the higher KPC expression (a 1.8-fold increase relative to SL-1A) combined with the additional porin loss from the *ompK36* partial deletion (19, 20).

### The evolution of carbon metabolism in sublineages 1 and 3.

**FIG 3:**
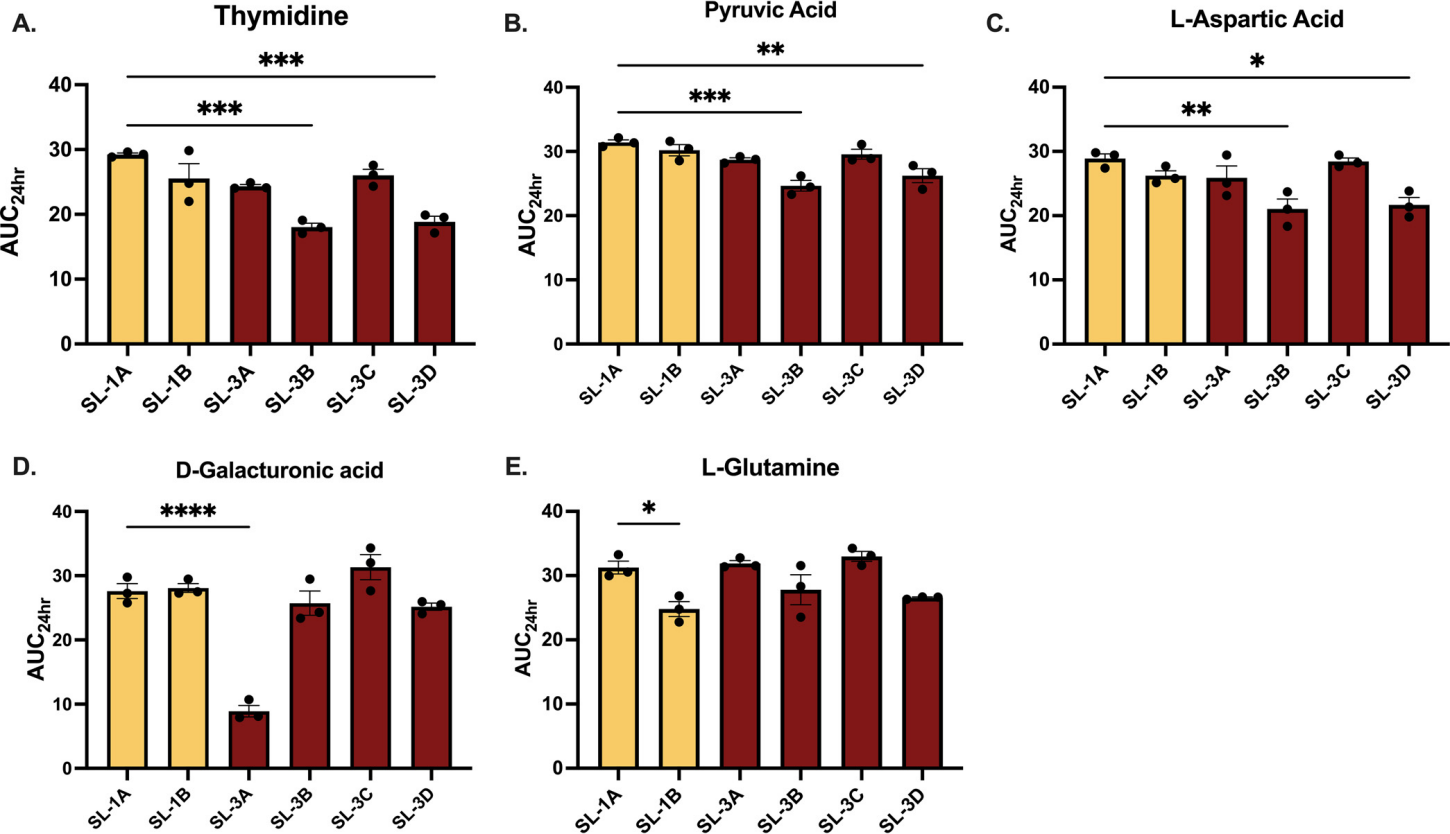
AUC_24_ for thymidine (A), pyruvic acid (B), l-aspartic acid (C), d-galacturonic acid (D), and l-glutamine (E) respiration curves from Biolog phenotypic microbial assays. Sublineage 1 is highlighted in yellow; sublineage 3 is highlighted in red. Adjusted culture (100-μL aliquots) in minimal medium plus tetrazolium dye were added to each well and incubated at 37°C for 24 h. Visualized substrates showed significance in AUC_24_ by one-way ANOVA (*, *P* < 0.05).

Metabolism has been shown to impact the development of resistance as well as virulence in bacteria, and we found that the majority of nonsynonymous mutations were acquired in metabolic genes (Table 3; see also Data Set S1) (21–24). Since many mutations affected genes involved in carbon metabolism, we tested whether the strains exhibited phenotypic changes in carbon metabolism that developed *in vivo* over time using Biolog Phenotype MicroArrays. When we compared the strains from sublineages 1 and 3 to the first isolated strain (SL-1A), we found decreases in carbon metabolism only in SL-3B and SL-3D, which corresponded to decreased area under the curve at 24 h(AUC_24_) when grown with thymidine, l-aspartic acid, and pyruvic acid (Fig. 3 and Fig. S2). Singularly, the terminal strain in sublineage 1, SL-1B, had a significant decrease in l-glutamine metabolism compared to initial strain SL-1A, and SL-3A had a significant decrease in d-galacturonic acid metabolism compared to initial strain SL-1A. Despite genetic changes, the strains’ overall abilities to utilize different carbon sources remained unaffected.

**TABLE 3:** Mutations acquired in metabolism- and virulence-associated genes as strains in sublineages 1 and 3 evolved

Gene category or gene	AA change	Function	Mutation presence^*a*^ in strain from:					
			Sublineage 1		Sublineage 3			
			SL-1A	SL-1B	SL-3A	SL-3B	SL-3C	SL-3D
Metabolism								
*pykF*	M23K	Pyruvate kinase; step 5 of pyruvate synthesis from d-glyceraldehyde 3-phosphate			●	●	●	●
*add*	Δ	Adenosine deaminase; purine catabolism				●	●	●
*gapdh*	Δ	Glyceraldehyde-3-phosphate dehydrogenase				●	●	●
*dtpA*	P171A	Dipeptide/tripeptide permease A; proton-dependent transport of antibacterial tripeptides, controlled by OmpR					●	●
*xylB*	T317M	Pentose kinase (xyulokinase)						●
*exuT*	P152Q	Hexuronate transporter			●			
*lacZ*	Y168H	Beta-d-galactosidase		●				
Virulence								
*envZ*	L88F	Osmolarity two-component sensor histidine kinase; porin regulation and type 3 fimbriae regulation important for biofilm formation			●	●	●	●
*rcsC*	G855V	Hybrid sensor histidine kinase/response regulator; regulates capsule and fimbriae production important for biofilm formation			●	●	●	●
*mviM*	E132K	Virulence factor MviM; oxidoreductase necessary for murein synthesis				●	●	●
*wzc*	I402S	Tyrosine kinase in capsule operon				●	●	●
*wzc*	G565S	Tyrosine kinase in capsule operon		●				

a ●, Mutation present.

10.1128/msphere.00190-22.1 DATA SET S1 Core genome mutations present in all 10 carbapenem-resistant Klebsiella pneumoniae (CRKP) strains isolated from patient X, identified by the *breseq* algorithm. Download Data Set S1, XLSX file, 0.03 MB. Copyright © 2022 Kalu et al. 2022 Kalu et al. https://creativecommons.org/licenses/by/4.0/ This content is distributed under the terms of the Creative Commons Attribution 4.0 International license.

10.1128/msphere.00190-22.5 FIG S2 Respiration curves for thymidine, pyruvic acid, l-aspartic acid, d-galacturonic acid, and l-glutamine from Biolog phenotypic microbial assays for strains in sublineages 1 and 3. Negative-control well included the Biolog IF0a medium with the redox dye and no bacterial culture. Plates were grown at 37°C for 24 h. Download FIG S2, TIF file, 0.10 MB. Copyright © 2022 Kalu et al. 2022 Kalu et al. https://creativecommons.org/licenses/by/4.0/ This content is distributed under the terms of the Creative Commons Attribution 4.0 International license.

### The evolution of biofilm production in sublineages 1 and 3.

**FIG 4:**
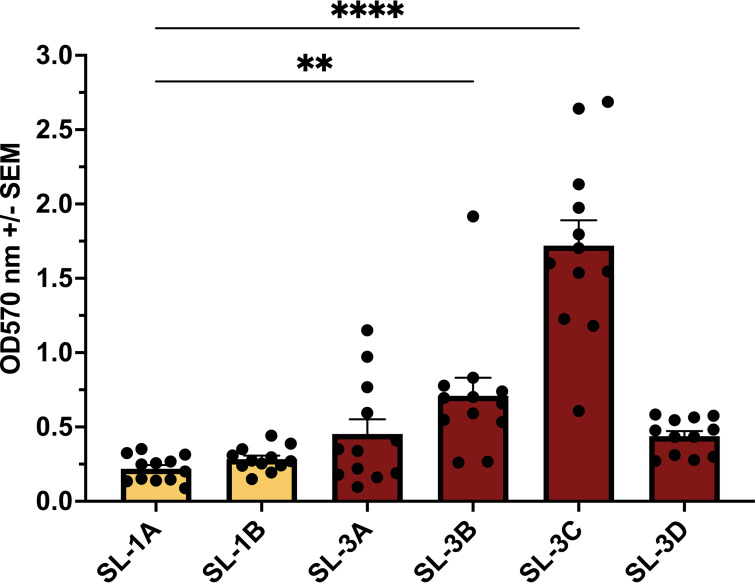
Biofilm production over time by the strains in sublineages 1 and 3. Sublineage 1 is highlighted in yellow; sublineage 3 is highlighted in red. Biofilm production quantified by degree of crystal violet dye at an optical density at 570 nm (OD_570_). Significance by one-way ANOVA (*, *P* < 0.05).

The strains also acquired mutations in genes specifically related to biofilm production. Notably, the terminal strain of sublineage 1, SL-1B, acquired a nonsynonymous mutation in the *wzc* gene (G565S), which encodes a tyrosine kinase involved in capsule production. The mutation affects the cytoplasmic region of the protein and has been documented to cause increased capsule production and decreased biofilm formation (25). In contrast, three of the strains in sublineage 3 acquired a different mutation in *wzc* (I402S). The effect of the I402S mutation on capsule production has not been characterized yet as it occurs in a highly conserved G-rich domain with unknown function in the periplasmic region of the protein (26). Additionally, all the strains in sublineage 3 acquired nonsynonymous mutations in genes encoding the two-component system sensor kinases EnvZ and RcsC. EnvZ, together with the transcriptional regulator OmpR, regulates the expression of porins and type 3 fimbriae, which are important components of biofilm (27, 28). RcsC, together with the transcriptional regulator RcsB, regulates capsule production, which is also important for biofilm production (29). In addition, three of the four strains in sublineage 3, SL-3B, SL-3C, and SL-3D, also developed mutations in *mviM*, which produces an oxidoreductase involved in murein synthesis (30). To assess changes in biofilm production across the evolved strains, we measured biofilm growth using a microtiter assay. We observed that two of these isolates (SL-3B and SL-3C) produced more biofilm than the first isolate collected (Fig. 4). Relative to the initial strain (SL-1A), SL-3C had the highest biofilm production, measuring nearly 7-fold higher.

### Exposure to meropenem at subinhibitory concentrations increases biofilm production.

**FIG 5:**
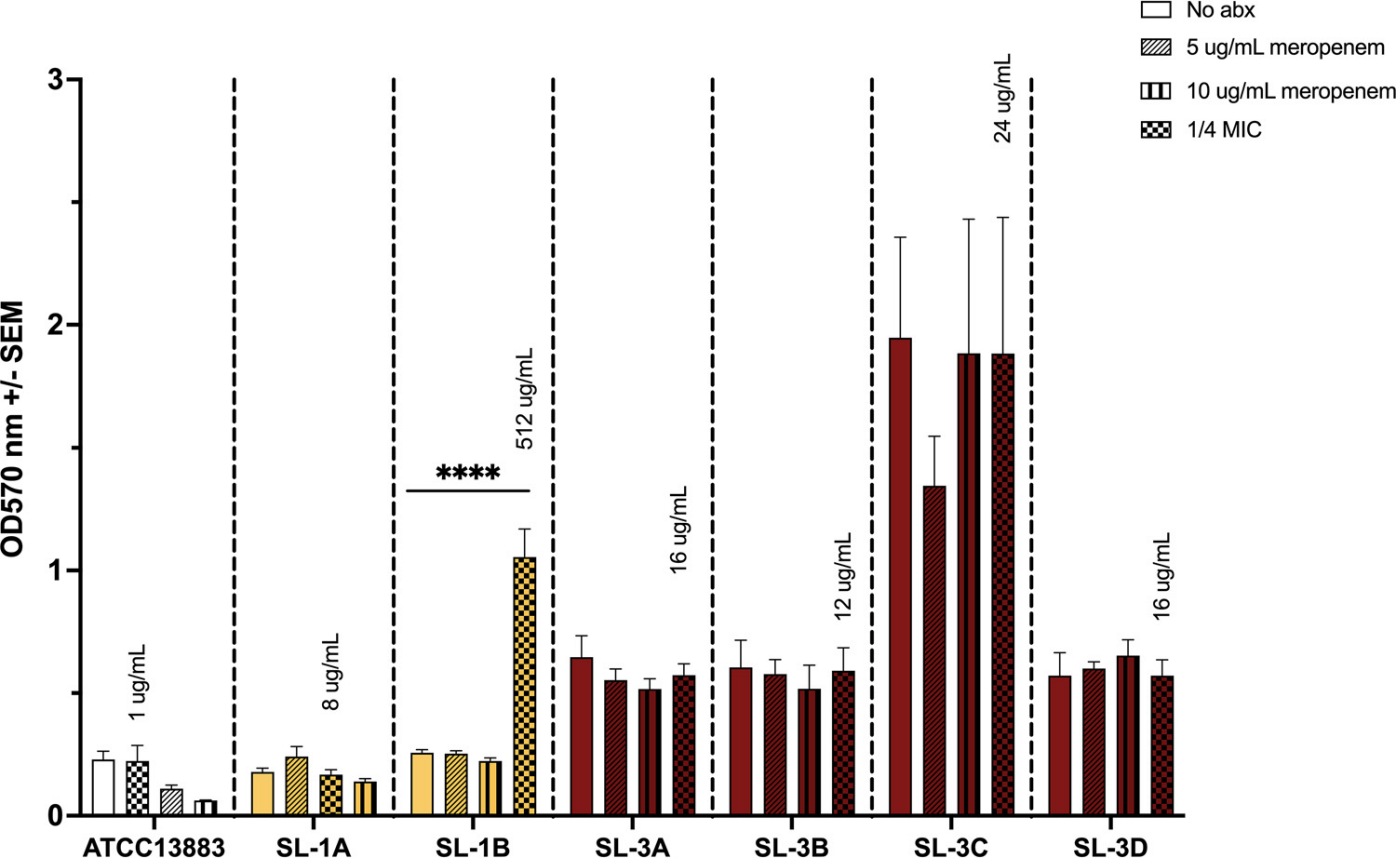
Biofilm production after 24 h of sub-MIC meropenem exposure. Sublineage 1 is highlighted in yellow; sublineage 3 is highlighted in red. Crystal violet assay was performed after 24 h of exposure. Significance by one-way ANOVA (*, *P* < 0.05). The package insert for meropenem states that urine concentrations remain above 10 μg/mL for up to 5 h after a single 500-mg dose, and a previous study stated that meropenem is present in the urine at levels above 1 μg/mL for 12 h regardless of dosing. Thus, the bacteria were grown under the following conditions: no antibiotic (no abx), meropenem at 5 μg/mL, 10 μg/mL, and one-quarter MIC.

We next examined the impact of meropenem exposure at subinhibitory concentrations on biofilm production in these strains, as increases in biofilm production have been documented in response to antibiotic exposure (31, 32). The package insert for meropenem states that urine concentrations remain above 10 μg/mL for up to 5 h after a single 500-mg dose, and a previous study showed that meropenem is present in the urine at levels above 1 μg/mL for 12 h regardless of dosing (33, 34). Thus, we grew the bacteria under the following conditions: no antibiotic, meropenem at 5 μg/mL, 10 μg/mL, and one-quarter MIC. We also performed a 24-h growth curve which showed no change in growth in medium containing these concentrations of meropenem (data not shown). The crystal violet biofilm assay was performed after 24 h of incubation. Interestingly, exposure to meropenem at one-quarter MIC induced a significant increase in biofilm formation in the terminal strain SL-1B in sublineage 1 (Fig. 5). On the other hand, another colonizing strain (SL-3C) in sublineage 3, which produced the strongest biofilm among the evolved strains at baseline, maintained the same level of biofilm production under sub-MIC exposure to meropenem. The initial strain SL-1A, as well as the infected strains from sublineage 3 (SL-3A, SL-3B, and SL-3D), did not show significant increases in biofilm production in response to sub-MIC meropenem exposure.

### Correlations with antibiotic therapy and clinical course of Patient X.

As meropenem exposure increased along with genetic and phenotypic changes, we investigated the relationship between meropenem therapy in patient X and the phenotypic changes in the strains in sublineages 1 and 3 by performing a correlation matrix using Pearson’s correlation (Fig. S3). Cumulative increases in days of meropenem therapy compared to increases in *bla*_KPC_ expression and biofilm production over time resulted in *R* values of 0.67 and 0.37, respectively (*P* = 0.144 and 0.470, respectively). A trend between meropenem days of therapy and decreases in l-aspartic acid, thymidine, and pyruvic acid metabolism was observed with *R* values of −0.63, −0.74, and −0.67, respectively (*P* = 0.176, 0.089, and 0.145, respectively).

10.1128/msphere.00190-22.6 FIG S3 Correlation matrix of meropenem days of therapy (DOT), KPC expression, biofilm production, and carbon source utilization for strains in sublineages 1 and 3. Red denotes a positive correlation and direct relationship; yellow denotes a negative correlation and inverse relationship. Phenotypic markers with correlations to meropenem therapy closest to 1.00 or −1.00 and *P* values of less than 0.20 are highlighted in orange and starred. Download FIG S3, TIF file, 1.0 MB. Copyright © 2022 Kalu et al. 2022 Kalu et al. https://creativecommons.org/licenses/by/4.0/ This content is distributed under the terms of the Creative Commons Attribution 4.0 International license.

## DISCUSSION

Antibiotic exposure can drive host-adapted evolution of bacteria through the acquisition of mutations that affect antibiotic resistance, metabolism, and virulence (21, 35–37). Here, we examined the long term in-human evolution of antibiotic resistance, metabolism, and biofilm production in urinary CRKP strains isolated from patient X over 4.5 years and correlated these adaptations with the patient’s clinical course and antibiotic exposure. Through WGS and mutational analysis, we showed that the K. pneumoniae strain that initially infected the patient evolved into two clades with three sublineages. The overlap of time in the sublineages suggests that the strains coexisted and coevolved in the patient’s bladder. We performed further analysis to identify the mutations that differed between the sublineages and discovered that the strains evolved mutations in genes related to antibiotic resistance, cell metabolism, and biofilm production. Strain SL-1A was the only strain that did not have the beta-lactamase gene *bla*_OXA-9_, suggesting that dynamic plasmid changes may have occurred that could have supported the changes in resistance and biofilm production. The changes correlate with times in which the patient received meropenem, colistin, and tigecycline therapies in 2010 and 2011, while in 2012 the patient received only meropenem and from 2013 to 2015 the patient received meropenem and tigecycline combination therapy. Lopatkin et al. showed that antibiotics do not have a direct impact on conjugation efficiency; however, antibiotics impact the population dynamics, which can promote or suppress plasmid exchanges in a given bacterial population (38). It is possible that the change in patient X’s antibiotic therapy after 2011 in combination with the structural changes to the bladder due to his age and comorbid conditions, such as benign prostatic hyperplasia, may have created a favorable environment for the plasmid exchanges between bacteria in the bladder to occur (39).

We also discovered differences between the sublineages in meropenem resistance, carbon metabolism, and biofilm production in comparison to the earliest strain (SL-1A) as they progressed through time. Patient X was prescribed meropenem-containing regimens throughout his clinical course, and we identified mutations in porin genes that have previously been shown to directly impact and support high-level carbapenem resistance (19, 20). We observed that the more evolved CRKP strain (SL-1B) exhibited higher *bla*_KPC_ expression, with a corresponding higher meropenem MIC, relative to the earlier strain SL-1A. SL-1B had mutations in both porins OmpK35 and OmpK36 that, in combination with the KPC-3 enzyme, likely contributed to the highest meropenem MIC (>1,024 μg/mL) observed, although this would need to be confirmed in future work. Several studies have shown that loss of one or both porins significantly increases carbapenem MICs in K. pneumoniae (19, 20, 40–43). The partial deletion of the *ompK36* gene in SL-1B may have arisen during the 10-day course of high-dose meropenem therapy (totaling 2,000 mg per day) the patient received a few days prior to isolation of the CRKP strain. The consistent pressure of meropenem therapy may have catalyzed the porin loss and the increase in meropenem MIC.

In addition to changes in meropenem resistance, we also observed altered metabolism in sublineages 1 and 3 using Biolog Phenotype MicroArrays. Strains SL-3B and SL-3D in sublineage 3 exhibited decreases in l-aspartic acid, thymidine, and pyruvate metabolism. Although we identified mutations in *pykF* and *gapdh* involved in glycolysis in SL-3B and SL-3D, these mutations were not specific to SL-3B and SL-3D and thus did not fully explain the change in metabolism. Similarly, SL-3B, SL-3C, and SL-3D acquired deletions of the *add* gene. Even though all three strains have the deletion of the *add* gene, only SL-3B and SL-3D exhibited small decreases in adenosine metabolism, which were not statistically significant (data not shown). In contrast, we also observed strain-specific alterations in metabolism that could potentially be explained by the detected mutations. The terminal colonizing strain of sublineage 1, SL-1B, carried a deletion in *ompK36* which encodes a porin used to transport glutamine into the cell (44). The deletion in *ompK36* likely contributed to the small but significant decrease in glutamine metabolism observed in SL-1B, although further studies are needed to confirm the contribution of OmpK36 to glutamine transport in these strains. The mutation in the gene for the hexuronate transporter ExuT in SL-3A could possibly explain the significant decrease in d-galacturonic acid metabolism seen only in that strain by limiting transport of d-galacturonic acid into the cell. Also, both SL-3B and SL-3D were isolated when the patient was displaying symptoms of infection. It is possible that the metabolic landscape of the bladder changed over time due to the episodes of infection and colonization and that only SL-3B and SL-3D developed changes in carbon utilization as compensatory response. Taken together, alterations in the utilization of those substrates suggest niche-specific adaptations of the strains to confer long-term survival in the dynamic environment of the bladder (45). Amino acid utilization is linked to amino acid transport in urine, and loss of dipeptide transport was shown to cause an inability to establish infection by urinary Escherichia coli strains in a murine bladder model (46). In addition, the loss of infection potential in gluconeogenic mutants of the urinary E. coli strain CFT073 suggests a preference of amino acids as an energy source in the bladder (47). However, there are no studies demonstrating the role of specific amino acid utilization in colonization of the mouse bladder by urinary bacteria (48).

Regarding biofilm formation, all of the strains in sublineage 3 acquired mutations in *envZ* and *rcsC*, genes encoding the two-component system kinases that are involved in regulating components of biofilm formation, such as porin, fimbriae, and capsule production (25–37, 49). These strains also had nonsynonymous mutations in *wzc*, encoding a tyrosine kinase involved in capsule production. Notably, while the more evolved strains in sublineage 3 (SL-3B, SL-3C, and SL-3D) all exhibited higher biofilm production relative to the earliest strain (SL-1A), the only colonizing strain in sublineage 3 (SL-3C) had the highest biofilm production of all the strains, consistent with published literature supporting the link between biofilm production and increased persistence in the bladder by allowing bacteria to adhere to and enter bladder cells to form intracellular communities that can cause reinfection (29, 45). The patient also had a chronic indwelling catheter present throughout the 4.5 years of infection and colonization episodes. Although the catheter was changed during each hospital admission, it is possible that the bacteria formed biofilm communities on the catheter and seeded the bladder (50). Biofilm production was similar for the infected strains (SL-3A and SL-3B) in sublineage 3 but increased sharply in SL-3C and then returned to similar levels to those of the earlier infected strains (SL-3A and SL-3B) for SL-3D as the patient exhibited renewed symptoms of infection in 2015. Interestingly, SL-3C was isolated from the patient when he was asymptomatic, while the other strains in sublineage 3 were isolated when the patient exhibited symptoms of a urinary tract infection.

Meropenem was the only antibiotic that was consistently given throughout the patient’s clinical course. Additionally, the increases in *bla*_KPC_ expression and meropenem MICs over time correspond with increases in the duration of meropenem therapy preceding the strain isolation. Patient X was given meropenem therapy at 1,000 mg intravenously twice a day for only 4 days when SL-1A was isolated. When each subsequent strain, except for SL-1B, was isolated, the patient received at least 4 days of meropenem therapy totaling 2,000 mg per day in addition to at least 4 days of tigecycline totaling 100 mg per day. Because the CRKP strains were often exposed to sub-MIC meropenem concentrations during treatment, we examined the impact of sub-MIC exposure of meropenem on biofilm production. We found that sub-MIC meropenem exposure minimally impacted biofilm production of infected strains but significantly induced or maintained high biofilm production in strains isolated from the patient in the absence of symptoms of infection (SL-1B and SL-3C, respectively). Sub-MIC antibiotic exposure has been shown to increase biofilm production in several pathogens, such as Pseudomonas aeruginosa and E. coli (32). A study published in 2019 also showed that concentrations of meropenem as low as 1/8 MIC caused up to a 15-fold induction of biofilm production in strains of Acinetobacter baumannii (31). To our knowledge, we are the first to demonstrate that sub-MIC meropenem exposure increased biofilm production in a carbapenem-resistant strain of K. pneumoniae during colonization but not infection, underscoring the need to limit unnecessary antibiotic exposure, especially in patients with chronic colonization with CRKP.

The terminal strains of sublineages 1 and 3 displayed genetic and phenotypic changes in meropenem resistance, metabolism, and biofilm production over time and were able to persist in the bladder of patient X for 4.5 years despite repeated meropenem exposure. We also showed that continued meropenem therapy was correlated with changes in KPC expression, biofilm production, and carbon metabolism, though the latter was not statistically significant.

Our study has several limitations. First, we only examined isolates from a single patient, which limits the broader applications of the study. In future work we plan to study whether similar correlations are observed over the course of chronic infection and colonization in additional patients. Also, we did not test the effects of specific mutations on the phenotypes we measured. If similar mutations are detected in KPC strains infecting other patients, then it will be important to test the effects of the specific mutations using genetics. Another limitation is that although SL-1A is not the true ancestral strain of the infection, we compared all subsequent changes in the strains to SL-1A to describe which traits were dominant in strains at the end of the 4.5-year period compared to those in strains isolated at the beginning. We also did not study the impact of other comorbid conditions, such as benign prostatic hyperplasia, contributing to the patient’s immune response to the presence and persistence of CRKP strains in the bladder. Despite these limitations, the strains isolated from this case patient over a prolonged period offer insights into the long-term in-human evolution of CRKP strains representing the predominant clone ST258. These findings highlight the genetic and phenotypic flexibility of K. pneumoniae and its ability to adapt to the dynamic ecosystem within the human body to support prolonged colonization and recurring infections. Studies have shown how K. pneumoniae can evolve *in vivo* and *in vitro* within biofilms and exhibit increased antibiotic resistance, as well as the ability for CRKP strains to alter their capsule phenotypes over time, impacting biofilm production (51–53). Further study into which metabolic pathways and transcriptional changes influenced by carbapenems to significantly induce biofilm production and acquire resistance mutations could shed light on how repeated courses of carbapenem therapy potentially drive long-term persistence of CRKP in the bladder.

### Conclusion.

We have offered here unique insights into the long-term in-human evolution of CRKP through multiple genetic and phenotypic adaptations affecting carbon metabolism, carbapenem resistance, and biofilm production to support chronic colonization and intermittent urinary tract infections. Our findings have important implications for antimicrobial stewardship, as repeated courses of carbapenem therapy in recurrent UTIs appeared to select for more robust biofilm production and higher KPC gene expression in CRKP accompanied by altered utilization of specific carbon substrates present in urine. A better appreciation of adaptations that support increased expression of resistance and virulence characteristics promises to open up new avenues for novel therapeutics that target niche-specific metabolic pathways of bacteria to combat antibiotic resistance.

## MATERIALS AND METHODS

### Case patient.

Patient X’s medical record was retrospectively reviewed to obtain pertinent demographic, laboratory, clinical data, and antimicrobial treatment details. As no interventions were made, informed consent was waived. A hospital encounter was defined as any visit to the hospital’s emergency department or inpatient admission. Each hospital encounter was detailed for time interval since initial hospitalization, length of hospital stays, antimicrobial agent(s) prescribed with the corresponding MICs for the CRKP isolates, and disposition location. Infection and colonization designations were given per documentation in the medical record by the treating physician and in accordance with the CDC/NHSN 2008 surveillance definitions (54). Infection was defined as a urine culture positive for bacteria with at least one symptom such as fever, dysuria, or frequency. Colonization was defined as a urine culture positive for bacteria with no clinical signs or symptoms (54). All data were managed with the Research Electronic Data Capture (REDCap) software hosted at the University of Southern California, a secure web-based application designed to support data capture for research studies (55).

### Study isolates.

Ten CRKP isolates were collected from the patient’s urine culture over a 4.5-year period during hospital admissions and cryopreserved at −80°C as part of the longitudinal surveillance program to evaluate antimicrobial resistance trends at the institution. Study strains were grown in lysogeny broth (LB Lennox, Sigma) at 37°C with shaking. Broth microdilution susceptibility testing to meropenem (Sigma) was performed in triplicate in cation-adjusted Mueller-Hinton broth (Sigma) per CLSI guidelines (56) using a modified concentration range of 4, 8, 16, 32, 48, 64, 96, 128, 256, 512, and 1,024 μg/mL to obtain more precise MICs for downstream experiments.

### Whole-genome sequencing.

K. pneumoniae genomic DNA was isolated from overnight cultures using the QIAmp DNA minikit (Qiagen). A DNA library was prepared using the Nextera XT DNA library preparation kit (Illumina) per the manufacturer’s instructions. The genomes were sequenced using an Illumina MiSeq instrument. *De novo* assembly, annotation, and MLST were performed using PATRIC with default options (57, 58). Trimmed reads were aligned to a ST258 clinical strain, AR_0113, from the Centers for Disease Control and Prevention (CDC) and the Food and Drug Administration (FDA) Antimicrobial Resistance Bank. Mutations were identified and compared using *breseq* (59). A maximum likelihood neighbor-joining phylogenetic tree was created using single-nucleotide polymorphisms (SNPs) in CLC Genomics Workbench 12.0.3. An SNP distance table was generated using the Center for Genomic Epidemiology phylogeny pipeline (60). A time-scaled phylogeny was constructed by Bayesian analysis using BEAST (v 1.10.4) (61). Clock rates of evolution and the time to the most recent common ancestor (MRCA) were used to construct the tree. A Jukes-Cantor model with estimated base frequencies and uncorrelated relaxed clock type with log-normal distribution and constant size of tree prior were also used. The chain was run for 100 million generations and sampled every 1,000 generations. A burn-in of 10 million states was removed from the run. The final tree was annotated with figtree (https://github.com/rambaut/figtree/).

### KPC gene expression.

To measure gene expression of *bla*_KPC_ by quantitative RT-PCR, overnight cultures of all 10 strains were adjusted to an optical density at 600 nm (OD_600_) of 0.05, then diluted by 1:10 and incubated to the mid-exponential phase in LB Lennox broth. Total RNA was extracted using the PureLink RNA minikit per the manufacturer’s instructions (Invitrogen) and converted to cDNA using the iScript RT SuperMix (Bio-Rad). The *recA* and *rpoD* genes were used as reference genes to normalize mRNA expression in each strain. Control strains were AR_0113 (KPC positive) and ATCC 13883 (KPC negative). qRT-PCRs were performed using SsoAdvanced Universal SYBR green Supermix (Bio-Rad) in a 10-μL reaction mixture as follows: 95°C for 3 min, 95°C for 30 s, annealing at 60°C for 30 s, and 72°C for 30 s for 35 cycles. The experiment was performed in triplicate.

### Phenotypic characterization of resistance, biofilm production, and metabolism.

**(i) Porin extraction and visualization using SDS-PAGE.** Porin extractions were performed on overnight cultures adjusted to an OD_600_ of 0.2 and grown for 1 h using sonication with 10 mM HEPES (Cytiva) and 2% *N*-lauroylsarcosine (Sigma) suspended in 10 mM HEPES as previously described (19). Porin extracts (0.25 μg of protein), as well as the PageRuler prestained protein ladder, 180 kDa (Thermo Scientific), were loaded into a 4 to 20% acrylamide gradient gel (Bio-Rad) and run at 70 V for 20 min, then at 110 V for 1 h using the Mini-Protean electrophoresis system (Bio-Rad). The gel was then stained using SimplyBlue SafeStain (Invitrogen) and visualized per the manufacturer’s instructions.

**(ii) Biofilm production.** For all 10 clinical strains, a 96-well flat-bottomed polyethylene culture microtiter dish was filled with 180 μL of LB supplemented with 1% glucose and 20 μL of the overnight culture diluted to a final OD_600_ of 0.05. LB supplemented with 1% glucose was used as a negative control, while K. pneumoniae ATCC 13883 was used as a positive control. The cultures were incubated at 37°C for 18 h, and each well was washed three times with phosphate-buffered saline (PBS), dried for 1 h at 60°C, and stained for 15 min with 180 μL of 2% crystal violet. The dye was solubilized with 180 μL of 33% (vol/vol) glacial acetic acid, and the absorbance was measured at OD_570_. Each assay was performed with six replicates and repeated twice.

The impact of sub-MIC meropenem exposure on biofilm production was examined in six of the 10 selected strains and the control (lab strain ATCC 13883). Each strain was exposed to no antibiotic, one-quarter MIC, 5 μg/mL meropenem, and 10 μg/mL meropenem and tested in triplicate. Each well contained 100 μL of the various meropenem concentrations in LB supplemented with 1% glucose and 100 μL of the overnight culture diluted to a final OD_600_ of 0.05. Sterile LB supplemented with 1% glucose was used as a negative control. The plate was incubated at 37°C for 24 h of shaking at 200 rpm. Growth was tracked by measuring optical density (OD_600_) every 10 min in the Epoch2 microplate spectrophotometer (BioTek). The crystal violet assay was performed after 24 h using the same protocol as described above.

**(iii) Metabolic phenotype assays.** The carbon utilization profiles of the six strains from sublineages 1 and 3 were determined using Biolog Phenotype MicroArrays per the manufacturer’s instructions (62). Strains were precultured on LB agar plates at 37°C for 18 h. IF-0a fluid was inoculated with fresh colonies to reach an OD_600_ of 0.3, then diluted in 22 mL IF-0a and Dye Mix A. Biolog PM1 plates were inoculated with 100 μL of colonies diluted to an OD_600_ of 0.07 in IF-0a with Dye Mix A in each well and incubated at 37°C for 24 h in a double orbital shaker, and *A*_600_ was read every 10 min using a BioTek Epoch2 plate reader to measure oxidation of the tetrazolium redox-active dye as a proxy for growth. The resulting curves were analyzed, and the AUC_24_ was calculated using Growthcurver in RStudio (63). The experiment was performed in triplicate.

### Statistical analysis.

Summary statistics were performed using RStudio 1.3.959 (www.rstudio.com). The ordinary one-way analysis of variance (ANOVA) test was performed to analyze differences in relative *bla*_KPC_ expression, total biomass production, and AUC_24_ for each carbon source between study strains using Prism version 8.3.0 (GraphPad Software).

### Data availability.

Whole-genome sequencing (WGS) data for the 10 isolates used in this study are deposited in the NCBI database under BioProject number PRJNA791930.
