# Supplementary material for: In-Human Multiyear Evolution of Carbapenem-Resistant Klebsiella pneumoniae Causing Chronic Colonization and Intermittent Urinary Tract Infections: A Case Study
Source: mSphere. 2022 May 9;7(3):e00190-22. doi: 10.1128/msphere.00190-22 (PMC9241548; doi:10.1128/msphere.00190-22)
Supplement: TABLE S1 [file msphere.00190-22-s0002.docx]

**Table S1.** Complete description of all hospital stays and antibiotic therapy received by Patient X.

| Visit | Admit date | Length of stay | Infection/Colonization*^a^* | Empiric CRE therapy | Directed CRE therapy | Days of therapy |
| --- | --- | --- | --- | --- | --- | --- |
| 1 | 5/25/2010 | 25 | Infection | Colistin | Colistin | 17 |
|  |  |  |  |  | Tigecycline | 19 |
|  |  |  |  |  | Meropenem | 9 |
|  |  |  |  |  | Fosfomycin | 7 |
| 2 | 7/2/2010 | 12 | NC | Colistin | Colistin | 4 |
| 3 | 11/28/2010 | 3 | NC | Meropenem | Meropenem | 4 |
| 4 | 1/18/2011 | 3 | Colonization | Meropenem | Meropenem | 4 |
| 5 | 3/15/2011 | 13 | Infection | Meropenem | Meropenem | 13 |
|  |  |  |  |  | Colistin | 10 |
| 6 | 4/11/2011 | 7 | Infection | Colistin | Colistin | 5 |
|  |  |  |  | Meropenem | Meropenem | 5 |
|  |  |  |  |  | Tigecycline | 2 |
| 7 | 5/9/2011 | 6 | Infection | Meropenem | Meropenem | 6 |
| 8 | 9/9/2011 | 4 | Infection | Colistin | Colistin | 5 |
|  |  |  |  | Meropenem | Meropenem | 5 |
| 9 | 10/22/2011 | 3 | Colonization | Colistin | Colistin | 3 |
|  |  |  |  | Meropenem | Meropenem | 1 |
| 10 | 5/3/2012 | 2 | Colonization | Meropenem | Meropenem | 3 |
| 11 | 6/20/2012 | 3 | Infection | Meropenem | Meropenem | 3 |
| 12 | 7/3/2012 | 2 | NC | None |  |  |
| 13 | 7/18/2012 | 0 | Colonization | None |  |  |
| 14 | 8/7/2013 | 8 | Infection | Meropenem | Meropenem | 10 |
| 15 | 8/16/2013 | 0 | Colonization | None |  |  |
| 16 | 9/3/2013 | 9 | Colonization | Meropenem | Meropenem | 10 |
| 17 | 10/31/2013 | 4 | Infection | Meropenem | Meropenem | 5 |
|  |  |  |  |  |  |  |
| 18 | 2/18/2014 | 6 | NC | Meropenem | Meropenem | 6 |
|  |  |  |  | Tigecycline | Tigecycline | 6 |
| 19 | 3/7/2014 | 4 | Colonization | Meropenem | Meropenem | 5 |
|  |  |  |  | Tigecycline | Tigecycline | 5 |
| 20 | 9/3/2014 | 7 | Infection | Meropenem | Meropenem | 8 |
|  |  |  |  | Tigecycline | Tigecycline | 6 |
| 21 | 9/30/2014 | 7 | Infection | Meropenem | Meropenem | 9 |
|  |  |  |  | Tigecycline | Tigecycline | 8 |
| 22 | 11/12/2014 | 0 | Colonization | None |  |  |
| 23 | 11/27/2014 | 7 | Infection | Tigecycline | Tigecycline | 6 |
|  |  |  |  |  | Meropenem | 4 |
| 24 | 12/31/2014 | 9 | Infection | Meropenem | Meropenem | 10 |
|  |  |  |  | Tigecycline | Tigecycline | 10 |
|  |  |  |  | Neomycin bladder irrigation | | |
| 25 | 1/14/2015 | 4 | Infection | Amikacin | Amikacin | 3 |
|  |  |  |  | Meropenem | Meropenem | 4 |
|  |  |  |  | Tigecycline | Tigecycline | 4 |
|  |  |  |  | Neomycin bladder irrigation | | |

*^a^*Infection/Colonization designation based on CDC/NHSN 2008 surveillance definitions. “NC” denotes “no culture”, as no culture was obtained during that visit and the patient did not present with symptoms specific to a urinary tract infection.
