# Supplementary material for: In-Human Multiyear Evolution of Carbapenem-Resistant Klebsiella pneumoniae Causing Chronic Colonization and Intermittent Urinary Tract Infections: A Case Study
Source: mSphere. 2022 May 9;7(3):e00190-22. doi: 10.1128/msphere.00190-22 (PMC9241548; doi:10.1128/msphere.00190-22)
Supplement: TABLE S2 [file msphere.00190-22-s0003.docx]

**Table S2.** Single nucleotide polymorphism (SNP) distance matrix among the ten CRKP isolates isolated from Patient X calculated using CSI Phylogeny 1.1a web server.

|  | SL-2B | SL-2C | SL-2D | SL-3D | SL-1A | SL-2A | SL-3A | SL-1B | SL-3B | SL-3C |
| --- | --- | --- | --- | --- | --- | --- | --- | --- | --- | --- |
| SL-2B | 0 | 11 | 14 | 20 | 5 | 2 | 14 | 24 | 16 | 18 |
| SL-2C | 11 | 0 | 7 | 27 | 12 | 9 | 21 | 31 | 23 | 25 |
| SL-2D | 14 | 7 | 0 | 30 | 15 | 12 | 24 | 34 | 26 | 28 |
| SL-3D | 20 | 27 | 30 | 0 | 21 | 18 | 14 | 40 | 4 | 4 |
| SL-1A | 5 | 12 | 15 | 21 | 0 | 3 | 15 | 19 | 17 | 19 |
| SL-2A | 2 | 9 | 12 | 18 | 3 | 0 | 12 | 22 | 14 | 16 |
| SL-3A | 14 | 21 | 24 | 14 | 15 | 12 | 0 | 34 | 10 | 12 |
| SL-1B | 24 | 31 | 34 | 40 | 19 | 22 | 34 | 0 | 36 | 38 |
| SL-3B | 16 | 23 | 26 | 4 | 17 | 14 | 10 | 36 | 0 | 2 |
| SL-3C | 18 | 25 | 28 | 4 | 19 | 16 | 12 | 38 | 2 | 0 |
