# Supplementary figures and images for: In-Human Multiyear Evolution of Carbapenem-Resistant Klebsiella pneumoniae Causing Chronic Colonization and Intermittent Urinary Tract Infections: A Case Study
Source: mSphere. 2022 May 9;7(3):e00190-22. doi: 10.1128/msphere.00190-22 (PMC9241548; doi:10.1128/msphere.00190-22)

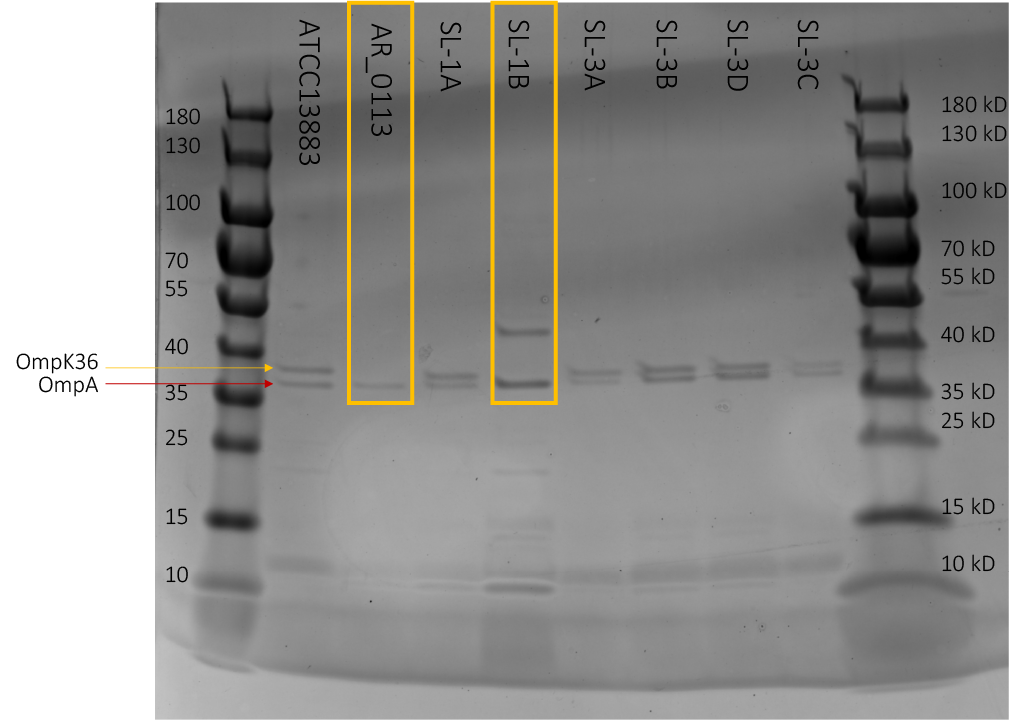

Supplement: FIG S1 [file msphere.00190-22-s0004.tif]

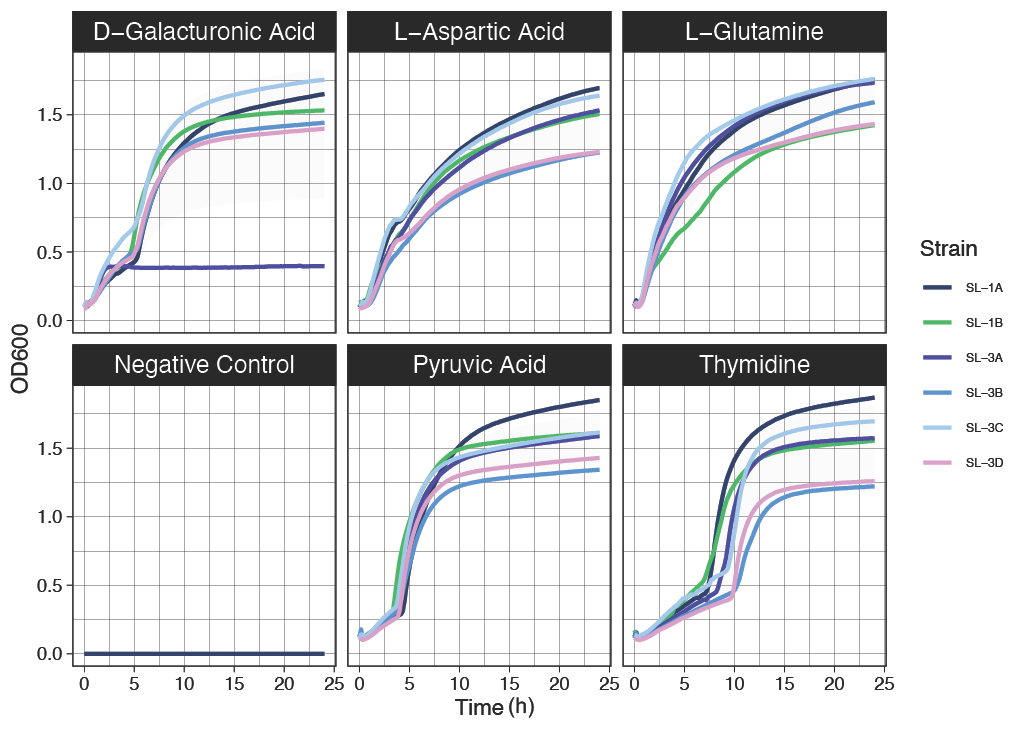

Supplement: FIG S2 [file msphere.00190-22-s0005.tif]

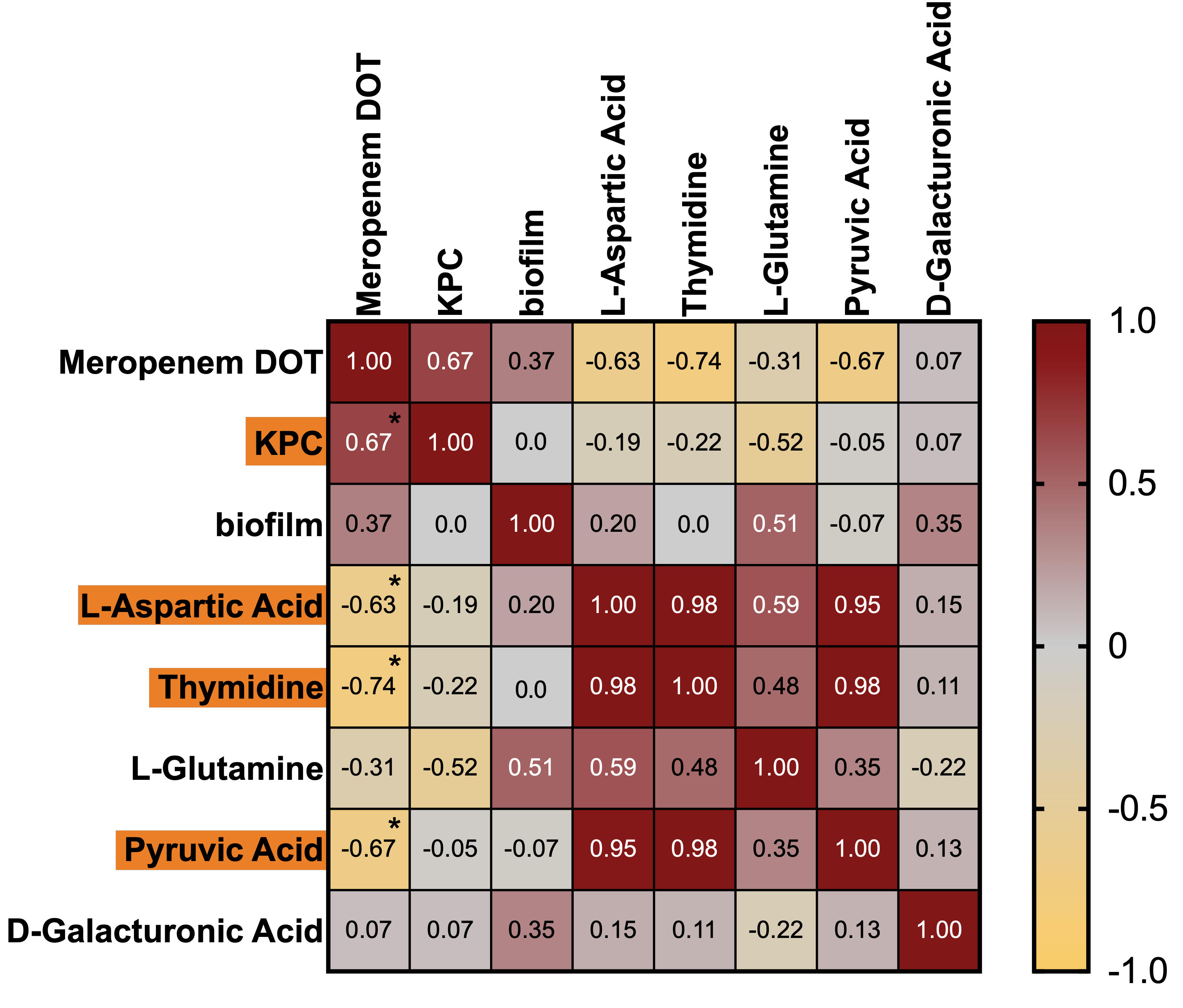

Supplement: FIG S3 [file msphere.00190-22-s0006.tif]
